# Electroplating Deposition of Bismuth Absorbers for X-ray Superconducting Transition Edge Sensors

**DOI:** 10.3390/ma14237169

**Published:** 2021-11-25

**Authors:** Jian Chen, Jinjin Li, Xiaolong Xu, Zhenyu Wang, Siming Guo, Zheng Jiang, Huifang Gao, Qing Zhong, Yuan Zhong, Jiusun Zeng, Xueshen Wang

**Affiliations:** 1 National Institute of Metrology (NIM), Beijing 100029, China; chenjian@nim.ac.cn (J.C.); xiaolong.xu@nim.ac.cn (X.X.); gsm@nim.ac.cn (S.G.); 18652908595@163.com (Z.J.); gaohf@nim.ac.cn (H.G.); zhongq@nim.ac.cn (Q.Z.); zhongyuan@nim.ac.cn (Y.Z.); 2 College of Metrology and Measurement Engineering, China Jiliang University, Hangzhou 310018, China; p20020854084@cjlu.edu.cn (Z.W.); jszeng@cjlu.edu.cn (J.Z.)

**Keywords:** transition edge sensors, bismuth, absorbers, electroplating deposition

## Abstract

An absorber with a high absorbing efficiency is crucial for X-ray transition edge sensors (TESs) to realize high quantum efficiency and the best energy resolution. Semimetal Bismuth (Bi) has shown greater superiority than gold (Au) as the absorber due to the low specific heat capacity, which is two orders of magnitude smaller. The electroplating process of Bi films is investigated. The Bi grains show a polycrystalline rhombohedral structure, and the X-ray diffraction (XRD) patterns show a typical crystal orientation of (012). The average grain size becomes larger as the electroplating current density and the thickness increase, and the orientation of Bi grains changes as the temperature increases. The residual resistance ratio (RRR) (*R*_300 K_/*R*_4.2 K_) is 1.37 for the Bi film (862 nm) deposited with 9 mA/cm^2^ at 40 °C for 2 min. The absorptivity of the 5 μm thick Bi films is 40.3% and 30.7% for 10 keV and 15.6 keV X-ray radiation respectively, which shows that Bi films are a good candidate as the absorber of X-ray TESs.

## 1. Introduction

A superconducting transition edge sensor (TES) is a detector with the superiority of high energy resolving ability, high quantum efficiency and a negligible dark-count rate. TES has been widely used in the field of X-ray detection [1,2]. X-ray TES is a very sensitive thermometer based on the transition between the superconducting and normal state of a superconducting metal film due to the thermal transfer from the absorber, which absorbs the energy of incident X-ray photons [3,4,5]. The energy resolution (Δ*E*) in the strong electro-thermal feedback is related to ~*C*^1/2^, *C* is the heat capacity of superconducting thin films and the absorber [6,7,8]. For the best Δ*E*, the *C* of the absorber should be as low as possible. Moreover, the absorber should show a high absorbing efficiency for X-ray radiation to realize high quantum efficiency [4]. Several kinds of metals, such as superconducting tantalum (Ta) [9,10] and tin (Sn) [11,12], normal metal Au [13,14], and semimetal Bi [15,16], have been used as absorbers. Among them, Bi and Au are widely used because of the high stopping X-ray power (high atomic number Z = 83 and Z = 79) and low specific heat [2,17]. Although Bi shows a smaller X-ray absorptivity than Au (35% vs. 52%, 5 μm thickness, 20 keV) [15], the specific heat is two orders of magnitude smaller, allowing for much larger collection area, which is beneficial for the aerospace X-ray detection [18]. 

Compared with the electroplating of Au films, which has been widely researched, research on Bi films are not so extensive. Brown et al. [19] compared the effect of electroplating and thermal evaporation methods on the grain structure of Bi films and the electroplated Bi with large grains showed a higher RRR. Gades et al. [15] examined the impact of the electroplating current density, agitation, film thickness, and seed layer thickness on the grain size, RRR and uniformity of Bi absorbers. Moral et al. [20] concluded the effects of electrodeposition parameters (electrolyte, deposition method, temperature and stirring) on morphology and electrical resistivity of the films. Sandnes et al. [21] concluded that the grain size increases with the increasing the film thickness, and Fabrega et al. [22] noted that grains with 1–10 μm in size could be obtained for a 4 μm Bi film, which depend on electroplating conditions. Lien et al. [23] found that the current density and stirring rate had a small effect on the crystal orientation, but pH was an important determinant of the crystal orientation at lower current densities. Costa et al. [24] found that the Bi films showed an irregular rough and polycrystalline structure grown in aqueous solutions, and the homogeneity, surface roughness, and density could be improved when the films grown in non-aqueous solvents.

Compared with the Bi absorbers obtained by thermal evaporation, the electroplated Bi could minimize the low-energy tails of X-ray TES and realized films with a thickness of micrometers [19,25]. As described in [21,24], the elemental Bi is mainly dissolved in oxidizing acids to form colorless Bi^3+^ cation. The Bi^3+^ is easy to form Bi_2_O_3_ precipitation even the pH rises very little. Therefore, a complex agent is needed to stabilize Bi^3+^ (polyhidroxy acids or alcohols). Moreover, a very low pH is better for the stabilization of the electroplating of Bi films. The composition of the electroplating solution will affect the morphology of the Bi films. In this work, we investigated the electroplating process of Bi films using the main solution of Bi^3+^ with glycerol, tartaric acid and potassium hydroxide as the additives [15,20,24]. The correlation of the electroplating current density and temperature with the morphology, grain structure, grain size, deposition rate and uniformity of the Bi films was studied. The RRR of the Bi films were measured using a liquid He system. The absorptivity of the electroplated Bi films with different thicknesses for 10 keV and 15.6 keV X-ray radiation were evaluated using a single-energy X-ray source system based on crystal diffraction.

## 2. Materials and Methods

### 2.1. Chemicals

All chemicals were used as obtained without any purification. Bismuth-nitrate pentahydrate (Bi(NO_3_)_3_·5H_2_O, 99.99%) and L(+)-Tartaric acid (2*R*,3*R*-(CHOHCOOH)_2_, 99.5%) were purchased from Aldrich. Concentrated nitric acid (HNO_3_, 70%), potassium hydroxide (KOH, 85%) and glycerol (99.5%) were obtained from Sigma-Aldrich. Water used in the experiments was deionized water.

### 2.2. Electroplating Process

The Bi films were deposited on a 4-inch silicon substrate in a commercial chip-scale electroplating system with the pump cycle of the electrolyte solutions and temperature controlling function (YAMAMOTO-MS, Tokyo, Japan) with a 4-inch chip clamping cathode and a Pt counter anode. A 125 nm Au layer with a 10 nm Ti adhesion layer made by a sputtering process served as the seed layer. The electroplating solution was composed by 1.35 M glycerol, 0.15 M bismuth (III) nitrate pentahydrate, 1.15 M potassium hydroxide, 0.33 M tartaric acid and 1.79 M nitric acid. The pH was ~0.3. To investigate the effect electroplating parameters on the quality of Bi films, the electroplating time was fixed at 2 min, the electroplating current densities were changed as 1, 2, 3, 5, 7, 9, 11, 13 and 15 mA/cm^2^, and the temperature of the electroplating solution varied to be 30 °C, 40 °C, 50 °C and 60 °C. Moreover, 5 μm Bi films were synthesized at different current densities to investigate the effect of the thickness to the grain size.

### 2.3. Characterization and Electrical Measurements

The morphology and grain size of the Bi films were characterized by a scanning electron microscope (SEM, FEI Helios NanoLab G3, FEI, Hillsboro, OR, USA). The thickness of the films was measured by a Bruker DektakXT (Bruker, Karlsruhe, Germany) step profile. The XRD pattern of Bi films was achieved by a Panalytical X’Pert PRO MPD diffractometer (Cu λ_Kα_ = 1.541874 Å, Malvern Panalytical Ltd., Malvern, United Kingdom) with the incidence angle fixed at 0.5°, and from 10° to 90° with a 2*θ* scanning speed of 0.5° per step. The sheet resistance was tested by mapping measurements with a CDE ResMap 178 system (Creative Design Engineering Inc., Cupertino, CA, USA) based on the van der Pauw method. The elements analysis was characterized by an X-ray photoelectron spectroscopy (XPS, ESCALAB 250Xi, Thermo Fisher Scientific, Waltham, MA, USA). The RRR was measured in a liquid Helium system with a probe and a source meter. The absorptivity of the electroplated Bi films with different thicknesses for 10 keV and 15.6 keV X-ray radiation were evaluated using a home-made single-energy X-ray source system based on crystal diffraction.

## 3. Results and Discussion

### 3.1. Morphology and Texture Study

**Figure 1:**
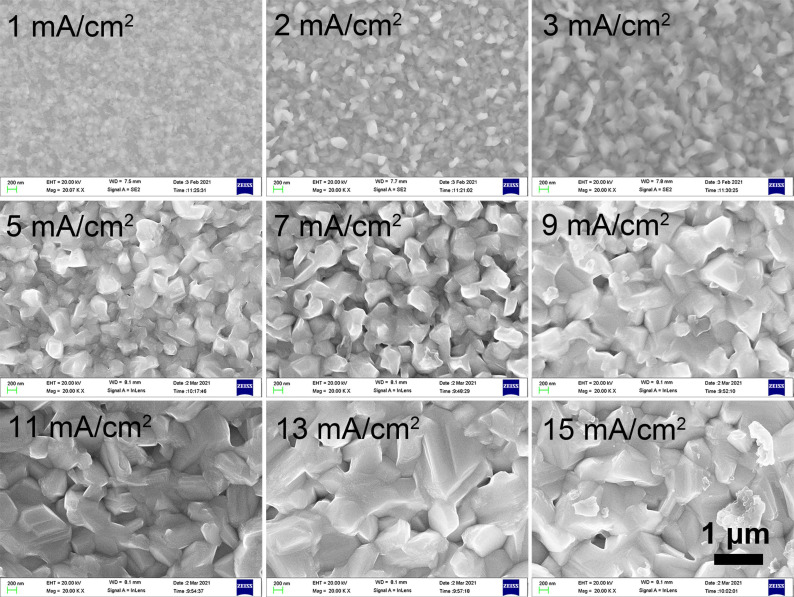
SEM images of the Bi films electroplated with different current densities at 30 °C for 2 min.

**Figure 2:**
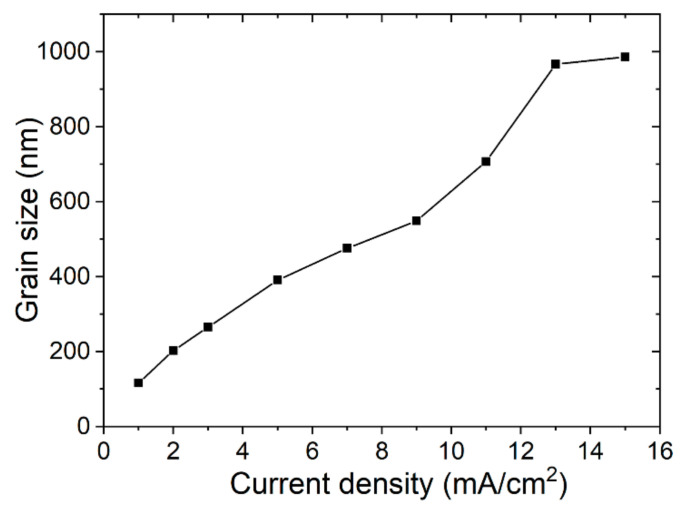
Average grain size achieved from the SEM images for different current densities.

The thicknesses of the Bi films are 92, 168, 266, 472, 632, 768, 892, 1012 and 1144 nm for the current densities of 1, 2, 3, 5, 7, 9, 11, 13 and 15 mA/cm^2^, respectively. The SEM images taken at the same magnification of the Bi films electroplated with different current densities (*J*_c_) at 30 °C for 2 min are shown in Figure 1, and the morphology changes obviously as the *J*_c_ rises. When the *J*_c_ is 1 mA/cm^2^, the Bi film shows compact grains with a small grain size and there is no obvious crystalline structure. The grains begin to grow and show a polyhedral structure as the *J*_c_ rises to 2–3 mA/cm^2^. As the *J*_c_ rises to 5–15 mA/cm^2^, the grains show a similar granular morphology with well-defined crystals and grow to an anisotropic rhombohedral structure [20]. Thus, *J*_c_ does have a significant influence on the grain structure of Bi films. The average grain sizes of Bi films are calculated by counting the grain size from the SEM images and are shown in Figure 2. As the *J*_c_ increases, the grain size becomes larger.

**Figure 3:**
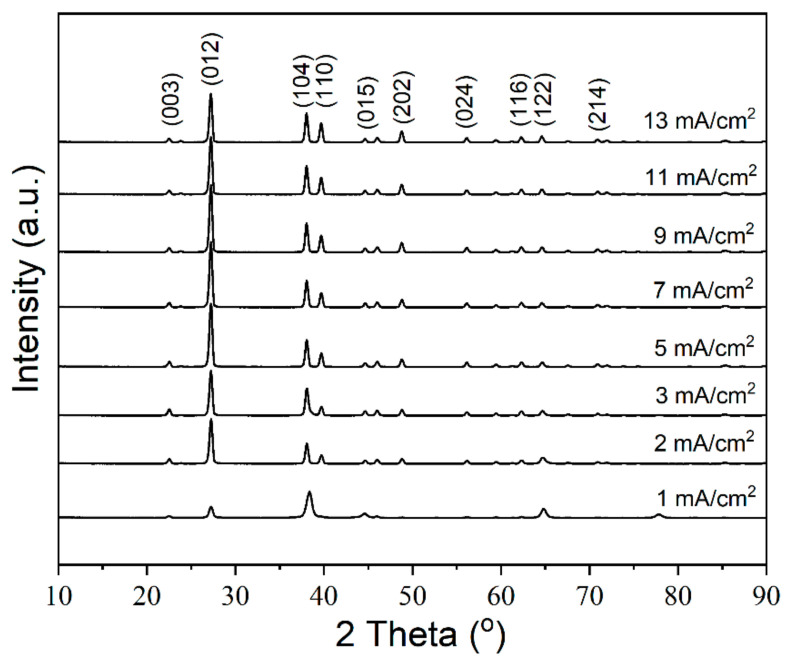
XRD patterns of Bi film electroplated with different current densities at 30 °C for 2 min.

The texture of the Bi films also varies with the *J*_c_. XRD patterns of the Bi films electroplated at different *J*_c_ are shown in Figure 3. All the samples exhibit a significant peak around 2*θ* = 27.1°, which is the presentation of a typical (012) plane of Bi (Bismuth, JCPDS card # 44-1246), and the most intense peak in the bulk polycrystalline Bi [20,21]. However, the intensity of (012) the peak of Bi films electroplated at 1 mA/cm^2^ is smaller than that electroplated at higher *J*_c_ and there is almost no (110) peak at 1 mA/cm^2^, which is consistent with the SEM results (Figure 1). The film shows compact Bi grains with the smallest grain size. However, when the *J*_c_ is > 2 mA/cm^2^, the (012) peaks become stronger and the (110) peaks are obvious, which indicates that the electroplating current density has a greater impact on the crystal orientation of Bi film, as the *J*_c_ is lower than 2 mA/cm^2^. However, the crystal orientation is not so much determined by the current density as the *J*_c_ is > 2 mA/cm^2^. The morphology of the polyhedral-like shape with different grain sizes is formed, which can be recognized as the rhombohedral form of Bi grains [26,27,28], as shown in Figure 1. All films show a pattern of polycrystalline and pure rhombohedral Bi [29], and the film structure and the main plane directions are dependent on the *J*_c_. The grains mainly grow along (012), (104) and (110) planes. In addition, there are no obvious peaks detected which could be attributed to the Bi_2_O_3._

**Figure 4:**
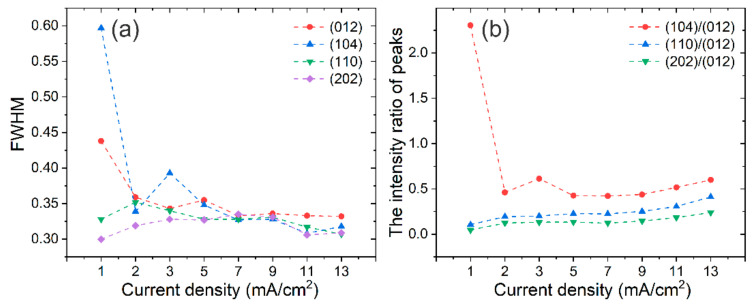
The FWHM of the main peaks (**a**) and the intensity ratio of peaks (**b**) obtained from XRD patterns.

Figure 4 shows the full width at half maximum (FWHM) and the intensity ratio of the peaks obtained from the XRD patterns. The FWHM of the main peaks do not change a lot as the *J*_c_ > 2 mA/cm^2^ (Figure 4a). The intensity ratio of (104)/(012), (110)/(012) and (202)/(012) increases slowly when the *J*_c_ is > 5 mA/cm^2^ (Figure 4b).

**Figure 5:**
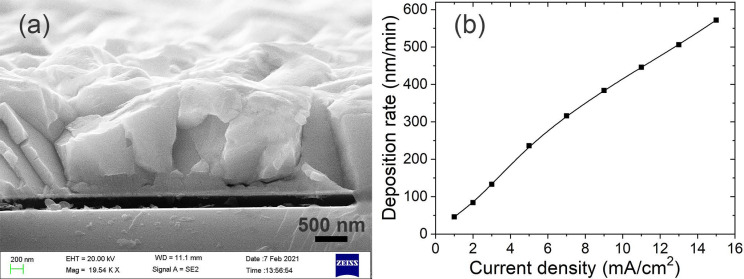
The SEM image of the cross-section of the Bi film electroplated with 9 mA/cm^2^ at 30 °C for 2 min (**a**) and the deposition rate of Bi films vs. current density (**b**).

Figure 5a shows the cross-section of the Bi film electroplated for 2 min with 9 mA/cm^2^ at 30 °C. The thickness of the Ti/Au seed layer is used as the reference to measure the thickness of the Bi films. As expected, the electroplating rate increases as a function of *J*_c_ from 46 nm/min at 1 mA/cm^2^ to 572 nm/min at 15 mA/cm^2^ as shown in Figure 5b.

**Figure 6:**
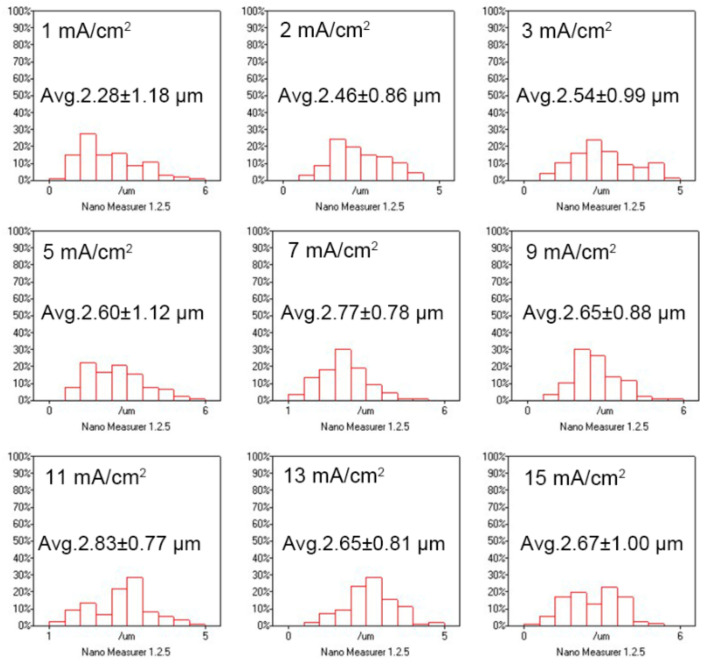
The distribution of grain size of 5 μm Bi films electroplated with different current densities at 30 °C.

To investigate the impact of the thickness of the Bi film on the grain size, 5 μm Bi films are electroplated at different current densities. As shown in Figure 6, the grain sizes are 2.28, 2.46, 2.54, 2.60, 2.77, 2.65, 2.83, 2.65, and 2.67 μm for the current density of 1, 2, 3, 5, 7, 9, 11, 13, and 15 mA/cm^2^, respectively. As the current density is > 3 mA/cm^2^, the grain size does not change obviously. Compared with the Bi films electroplated with the same current density at 30 °C for 2 min, the grain size increases obviously with the increasing film thickness.

**Figure 7:**
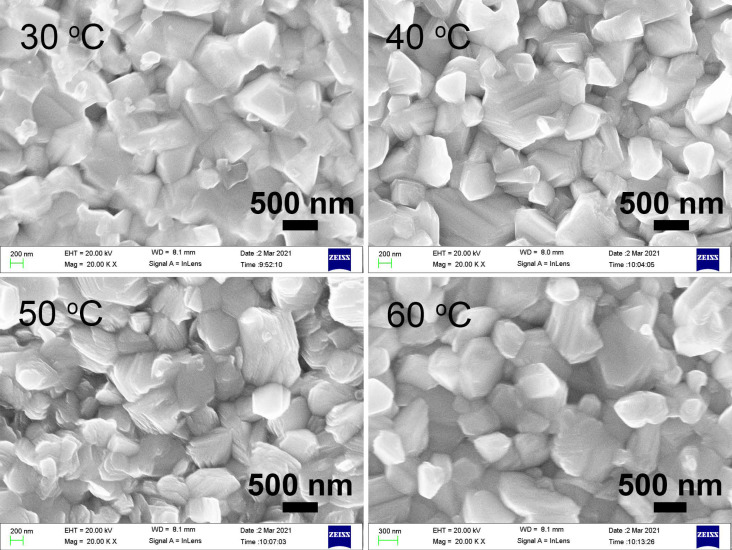
The SEM images of Bi films electroplated with different temperatures at 9 mA/cm^2^ for 2 min.

**Figure 8:**
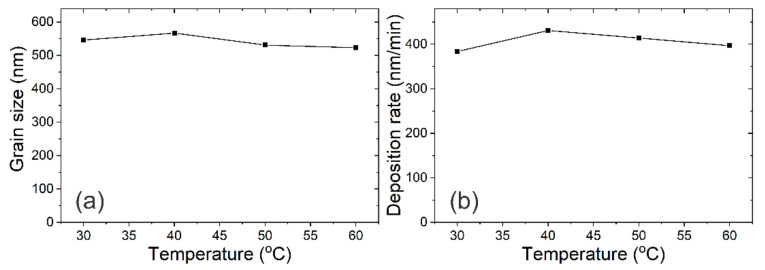
Average grain size obtained from SEM images (**a**) and the deposition rate vs. the electroplating temperatures (**b**).

The effect of electroplating temperature on the grain size and deposition rate of Bi films is also investigated. The electroplating time is fixed at 2 min and the current density is fixed at 9 mA/cm^2^, the thicknesses of the Bi films are 768, 862, 828, and 794 nm for Bi films electroplated at 30 °C, 40 °C, 50 °C, and 60 °C, respectively. Figure 7 shows the SEM images of Bi films electroplated from 30 °C to 60 °C. The grain shows an obvious multilayer structure from 40 °C and becomes loose. The average grain size and deposition rate is shown in Figure 8, and does not show significant change except that the ones at 40 °C show a little superiority.

**Figure 9:**
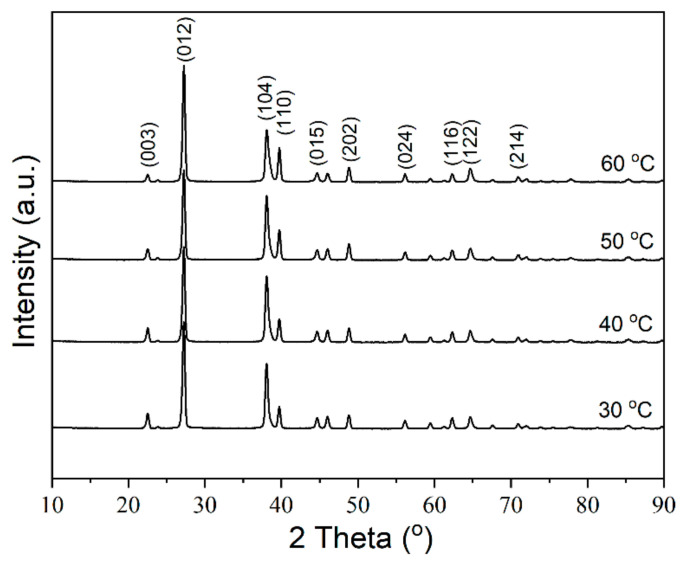
XRD patterns of Bi films deposited with different temperatures at 9 mA/cm^2^ for 2 min.

**Figure 10:**
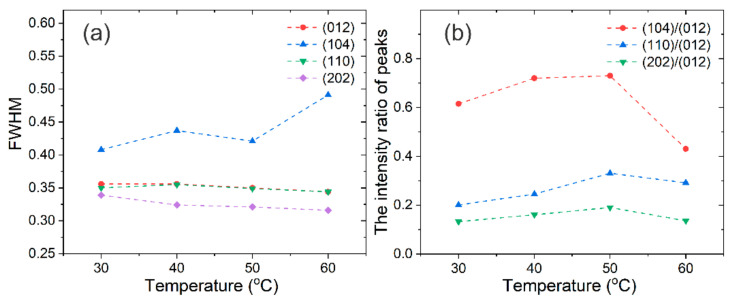
The FWHM of the main peaks (**a**) and the intensity ratio of the peaks (**b**) obtained from XRD patterns for different temperatures.

However, the preferential orientation of the Bi grain changes as the temperature rises, as shown in Figure 9. All the samples exhibit the significant peaks around 2*θ* at 27.1°, 37.9°, 39.6°, and 48.7°, which are assigned to the (012), (104), (110), and (202) reflections of Bi crystallites. Figure 10 shows the FWHM and the intensity ratio of peaks obtained from XRD patterns. The FWHM of the (012), (110) and (202) decrease with a small scale as the temperature rises and the FWHM of the (104) increases as the temperature rises (Figure 10a). The intensity ratio of (104)/(012), (110)/(012), (202)/(012) increases with the temperature before 50 °C and decreases at 60 °C. The electroplating temperature obviously affects the orientation of the Bi grains.

### 3.2. XPS Study

**Figure 11:**
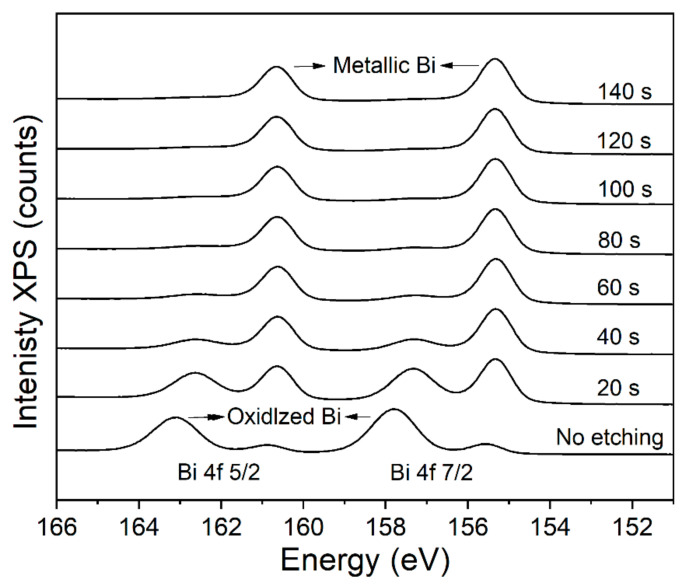
Bi 4f XPS spectra of the Bi film deposited with 9 mA/cm^2^ at 30 °C for 2 min.

To further investigate the composition of the Bi film electroplated with 9 mA/cm^2^ at 30 °C, XPS measurements are performed as shown in Figure 11. The XPS spectra with the depth of the Bi film are characterized by an in-situ etch process. At the surface, the peaks corresponding to the oxidized Bi (157.8 eV (Bi 4f_7/2_) and 163.1 eV (Bi 4f_5/2_)), and pure Bi (155.3 eV (Bi 4f_7/2_) and 160.6 eV (Bi 4f_5/2_)) are identified [20]. However, as the etch time increases, the oxidized Bi peaks become weaker and disappear after the accumulated 80 s etch process [30]. The etch rate is 0.25 nm/s, so the thickness of oxidized Bi is 15–20 nm, which indicates that only a small degree of oxidation occurs in ambient conditions and the Bi film is mostly composed by the pure Bi.

### 3.3. Electrical Properties

The electrical properties are also characterized. The sheet resistance of the Bi films electroplated with 9 mA/cm^2^ for 2 min at different temperatures are shown in Table 1. The sheet resistance decreases from 30 to 50 °C and increases at 60 °C. RRR (*R*_300 K_/*R*_4.2 K_) is related to the Bi grain size, and a large RRR is obtained for the electroplated Bi film with large grains. Therefore, the RRR behaves as a proof of the quality of electroplated Bi. RRR~1.0 has been reported as a benchmark reported in [19,20]. When measuring the RRR, the thickness of the seed layer will affect the measured value. A value of 1.44 (*R*_300 K_/*R*_4 K_) of a Bi film with the grain size 1 μm has been reported with 100 nm Au seed layer [15]. In this work, the seed layer is 125 nm Au on 10 nm Ti. The RRR (*R*_300 K_/*R*_4.2 K_) is 1.37 for the Bi film deposited with 9 mA/cm^2^ at 40 °C for 2 min and 1.57 for the Bi film deposited with 11 mA/cm^2^ at 30 °C for 2 min. The large RRR indicates that the electroplated Bi film shows a high quality.

### 3.4. X-ray Stopping Power

Bi is a good candidate as an absorber material for X-ray TES, because it shows a large X-ray stopping power as a high-Z element and a smaller specific heat [31]. The absorptivity for different thicknesses of Bi films electroplated with 9 mA/cm^2^ at 30 °C is characterized for 10 keV and 15.6 keV X-ray using a single-energy X-ray source system and summarized in Table 2. As the thickness increases, the absorptivity rises, and the absorptivity decreases as the energy becomes larger.

## 4. Conclusions

An electroplating deposition process is developed to fabricate Bi films as the absorbing layer for X-ray TESs. The effect of electroplating current density and temperature on the morphology, crystalline structure, and electrical properties are characterized. The Bi grains show a polycrystalline rhombohedral structure, with a typical crystal orientation of (012). The average grain size becomes larger as the electroplating current density and film thickness increase. The electroplating temperature does not obviously affect the grain size, and the orientation of the Bi grains begins to change when the temperature increases. High quality Bi films (thicknesses, 431 nm) with large grains (567 nm) and a high RRR (1.37) have been achieved with 9 mA/cm^2^ at 40 °C. The absorptivity of the 5 μm Bi films is 40.3% and 30.7% for the single-energy X-ray at 10 keV and 15.6 keV, which indicates that Bi films shows a greater superiority as the absorber in the X-ray TES devices.

## Figures and Tables

**Table 1:** The sheet resistance of Bi films electroplated with different temperatures at 9 mA/cm^2^ for 2 min.

Temperature (°C)	30	40	50	60
Average (Ω/*R*)	1.1234	0.9800	0.9600	1.0302

**Table 2:** The absorptivity of Bi films for X-ray at 10 keV and 15.6 keV.

Stopping Power	2 μm Bi	3 μm Bi	4 μm Bi	5 μm Bi
10 keV	12.691%	23.855%	31.524%	40.300%
15.6 keV	12.091%	19.868%	24.808%	30.689%

## Data Availability

The data presented in this study are available on request from the corresponding author.

## References

[1] Irwin K.D., Hilton G.C. (2005). Transition-Edge Sensors. Top. Appl. Phys..

[2] Ullom J.N., Bennett D.A. (2015). Review of superconducting transition-edge sensors for X-ray and gamma-ray spectroscopy. Supercond. Sci. Technol..

[3] Gottardi L., Nagayashi K. (2021). A Review of X-ray Microcalorimeters Based on Superconducting Transition Edge Sensors for Astrophysics and Particle Physics. Appl. Sci..

[4] Hummatov R., Adams J.S., Bandler S.R., Barlis A., Beaumont S., Chang M.P., Chervenak J.A., Datesman A.M., Eckart M.E., Finkbeiner F.M. (2020). Quantum Efficiency Study and Reflectivity Enhancement of Au/Bi Absorbers. J. Low Temp. Phys..

[5] Yoon W., Adams J.S., Bandler S.R., Betancourt-Martinez G.L., Chiao M.P., Chang M.P., Chervenak J.A., Datesman A., Eckart M.E., Ewin A.J. (2017). Design and Performance of Hybrid Arrays of Mo/Au Bilayer Transition-Edge Sensors. IEEE Trans. Appl. Supercond..

[6] Miniussi A.R., Adams J.S., Bandler S.R., Chervenak J.A., Datesman A.M., Eckart M.E., Ewin A.J., Finkbeiner F.M., Kelley R.L., Kilbourne C.A. (2018). Performance of an X-ray Microcalorimeter with a 240 μm Absorber and a 50 μm TES Bilayer. J. Low Temp. Phys..

[7] Patel U., Divan R., Gades L., Guruswamy T., Yan D., Quaranta O., Miceli A. (2020). Development of Transition-Edge Sensor X-ray Microcalorimeter Linear Array for Compton Scattering and Energy Dispersive Diffraction Imaging. J. Low Temp. Phys..

[8] Marcel L.v.d.B., Daniel T.C., Alex L., Mark F.C., Troy W.B., Matthias A.F., Simon E.L. (2000). High-resolution hard X-ray and gamma-ray spectrometers based on superconducting absorbers coupled to superconducting transition edge sensors. Proc. SPIE.

[9] Irimatsugawa T., Hatakeyama S., Ohno M., Takahashi H., Otani C., Maekawa T. (2015). High Energy Gamma-Ray Spectroscopy Using Transition-Edge Sensor With a Superconducting Bulk Tantalum Absorber. IEEE Trans. Appl. Supercond..

[10] Ridder M.L., Khosropanah P., Hijmering R.A., Suzuki T., Bruijn M.P., Hoevers H.F.C., Gao J.R., Zuiddam M.R. (2016). Fabrication of Low-Noise TES Arrays for the SAFARI Instrument on SPICA. J. Low Temp. Phys..

[11] Damayanthi R.M.T., Ohno M., Hatakeyama S., Takahashi H., Otani C. (2013). Development of Bulk Superconducting-Absorber Coupled Transition-Edge Sensor Detectors for Positron Annihilation Spectroscopy. IEEE Trans. Appl. Supercond..

[12] Hatakeyama S., Ohno M., Damayanthi R.M.T., Takahashi H., Kuno Y., Maehata K., Otani C., Takasaki K. (2013). Development of Hard X-Ray and Gamma-Ray Spectrometer Using Superconducting Transition Edge Sensor. IEEE Trans. Appl. Supercond..

[13] Taralli E., Gottardi L., Nagayoshi K., Ridder M., Visser S., Khosropanah P., Akamatsu H., van der Kuur J., Bruijn M., Gao J.R. (2020). Characterization of High Aspect-Ratio TiAu TES X-ray Microcalorimeter Array Under AC Bias. J. Low Temp. Phys..

[14] Nagayoshi K., Ridder M.L., Bruijn M.P., Gottardi L., Taralli E., Khosropanah P., Akamatsu H., Visser S., Gao J.R. (2020). Development of a Ti/Au TES Microcalorimeter Array as a Backup Sensor for the Athena/X-IFU Instrument. J. Low Temp. Phys..

[15] Gades L.M., Cecil T.W., Divan R., Schmidt D.R., Ullom J.N., Madden T.J., Yan D., Miceli A. (2017). Development of Thick Electroplated Bismuth Absorbers for Large Collection Area Hard X-ray Transition Edge Sensors. IEEE Trans. Appl. Supercond..

[16] Yan D., Divan R., Gades L.M., Kenesei P., Madden T.J., Miceli A., Park J.-S., Patel U.M., Quaranta O., Sharma H. (2018). Microstructure Analysis of Bismuth Absorbers for Transition-Edge Sensor X-ray Microcalorimeters. J. Low Temp. Phys..

[17] Yan D., Divan R., Gades L.M., Kenesei P., Madden T.J., Miceli A., Park J.-S., Patel U.M., Quaranta O., Sharma H. (2017). Eliminating the non-Gaussian spectral response of X-ray absorbers for transition-edge sensors. Appl. Phys. Lett..

[18] Chervenak J.A., Adams J.M., Bailey C.N., Bandler S., Brekosky R.P., Eckart M.E., Ewin A.E., Finkbeiner F.M., Kelley R.L., Kilbourne C.A. (2012). Fabrication of Microstripline Wiring for Large Format Transition Edge Sensor Arrays. J. Low Temp. Phys..

[19] Brown A.D., Bandler S.R., Brekosky R., Chervenak J.A., Figueroa-Feliciano E., Finkbeiner F., Iyomoto N., Kelley R.L., Kilbourne C.A., Porter F.S. (2008). Absorber Materials for Transition-Edge Sensor X-ray Microcalorimeters. J. Low Temp. Phys..

[20] Moral-Vico J., Casañ-Pastor N., Camón A., Pobes C., Jáudenes R.M., Strichovanec P., Fàbrega L. (2019). Microstructure and electrical transport in electrodeposited Bi films. J. Electroanal. Chem..

[21] Sandnes E., Williams M.E., Bertocci U., Vaudin M.D., Stafford G.R. (2007). Electrodeposition of bismuth from nitric acid electrolyte. Electrochim. Acta.

[22] Fàbrega L., Camón A., Costa-Krämer J.L., Pobes C., Parra-Borderías M., Fernández-Martínez I., Jáudenes R., Cereceda P., Ceballos M.T., Barcons X. (2014). Towards Mo/Au based TES detectors for Athena/X-IFU. Proc. SPIE.

[23] Lien C.-H., Hu C.-C., Tsai Y.-D., Wong D. (2012). Preferred Orientation Control of Bi Deposits Using Experimental Strategies. J. Electrochem. Soc..

[24] Cereceda-Company P., Costa-Krämer J.L. (2018). Electrochemical Growth of Bismuth for X-ray Absorbers. J. Electrochem. Soc..

[25] Sun Y., Gleber S.-C., Jacobsen C., Kirz J., Vogt S. (2015). Optimizing detector geometry for trace element mapping by X-ray fluorescence. Ultramicroscopy.

[26] Qin X., Sui C., Di L. (2019). Influence of substrate temperature on the morphology and structure of bismuth thin films deposited by magnetron sputtering. Vacuum.

[27] O’Brien B., Plaza M., Zhu L.Y., Perez L., Chien C.L., Searson P.C. (2008). Magnetotransport Properties of Electrodeposited Bismuth Films. J. Phys. Chem. C.

[28] Fedotov A., Shendyukov V., Tsybulskaya L., Perevoznikov S., Dong M., Xue X., Feng X., Sayyed M.I., Zubar T., Trukhanov A. (2021). Electrodeposition conditions-dependent crystal structure, morphology and electronic properties of Bi films. J. Alloy. Compd..

[29] Shu Y., Hu W., Liu Z., Shen G., Xu B., Zhao Z., He J., Wang Y., Tian Y., Yu D. (2016). Coexistence of multiple metastable polytypes in rhombohedral bismuth. Sci. Rep..

[30] Rajamani A.R., Jothi S., Kumar M.D., Srikaanth S., Singh M.K., Otero-Irurueta G., Ramasamy D., Datta M., Rangarajan M. (2016). Effects of Additives on Kinetics, Morphologies and Lead-Sensing Property of Electrodeposited Bismuth Films. J. Phys. Chem. C.

[31] Yan D., Weber J., Morgan K., Wessels A., Bennett D., Pappas C., Mates J., Gard J., Becker D., Fowler J. (2021). Transition-Edge Sensor Optimization for Hard X-ray Applications. IEEE Trans. Appl. Supercond..

